# RETRACTION: Mutual Regulation of MDM4 and TOP2A in Cancer Cell Proliferation

**DOI:** 10.1002/1878-0261.13776

**Published:** 2024-12-03

## Abstract

**RETRACTION:** T. Liu, H. Zhang, S. Yi, L. Gu, and M. Zhou, “Mutual Regulation of MDM4 and TOP2A in Cancer Cell Proliferation,” *Molecular Oncology* 13, no. 5 (2019): 1047–1058, https://doi.org/10.1002/1878-0261.12457.

The above article, published online on 04 February 2019 in Wiley Online Library (wileyonlinelibrary.com), has been retracted by agreement between the journal Editor‐in‐Chief, Kevin Ryan; FEBS Press; and John Wiley & Sons Ltd. The retraction has been agreed upon following an investigation into concerns raised by a third party, which revealed inappropriate image panel duplications between this (Figures 3B, 4C, and 5C) and other articles published previously by an overlapping group of authors in a different scientific context. The authors were unable to provide a satisfactory explanation and the original raw data, which has led the editors to lose confidence in the data presented. Therefore, the editors consider the conclusions substantially compromised and are retracting the paper.

**RETRACTION:** T. Liu, H. Zhang, S. Yi, L. Gu, and M. Zhou, “Mutual Regulation of MDM4 and TOP2A in Cancer Cell Proliferation,” *Molecular Oncology* 13, no. 5 (2019): 1047–1058, https://doi.org/10.1002/1878-0261.12457.

The above article, published online on 04 February 2019 in Wiley Online Library (wileyonlinelibrary.com), has been retracted by agreement between the journal Editor‐in‐Chief, Kevin Ryan; FEBS Press; and John Wiley & Sons Ltd. The retraction has been agreed upon following an investigation into concerns raised by a third party, which revealed inappropriate image panel duplications between this (Figures 3B, 4C, and 5C) and other articles published previously by an overlapping group of authors in a different scientific context. The authors were unable to provide a satisfactory explanation and the original raw data, which has led the editors to lose confidence in the data presented. Therefore, the editors consider the conclusions substantially compromised and are retracting the paper.

